# *SPRED1* Is Downregulated and a Prognostic Biomarker in Adult Acute Myeloid Leukemia

**DOI:** 10.3389/fonc.2020.00204

**Published:** 2020-02-27

**Authors:** Rui Zhang, Yan Zhang, Xianglan Lu, Weihong Xu, He Wang, Wenbin Mo, Hui Pang, Rurong Tang, Shibo Li, Xiaojing Yan, Yan Li

**Affiliations:** ^1^Department of Hematology, The First Affiliated Hospital of China Medical University, Shenyang, China; ^2^Department of Pediatrics, University of Oklahoma Health Sciences Center, Oklahoma City, OK, United States; ^3^Department of Anesthesiology, The First Affiliated Hospital of China Medical University, Shenyang, China

**Keywords:** SPRED1, AML, non-APL, MAPK, MIR126

## Abstract

We report herein that Sprouty-Related EVH1 Domain-Containing Protein1 (*SPRED1*) is downregulated and a prognostic biomarker in adult acute myeloid leukemia (AML). We determined mRNA levels of *SPRED1* in the bone marrow mononuclear cells from adult patients, including 113 AMLs and 22 acute lymphoblastic leukemias (ALLs), as well as in 37 healthy control subjects. Significantly decreased *SPRED1* mRNA expression was found in AML patients comparing to those in ALL patients and healthy controls, which was confirmed by immunocytochemistry analysis of SPRED1 protein and ELISA measurement of serum SPRED1 level. Further analysis demonstrated that *SPRED1* expression was significantly higher for most patients at complete remission after induction treatment than at diagnosis. Moreover, SPRED1 expression was significantly downregulated in M2 and M3 types. Non-acute promyelocytic leukemia (non-APL) patients with decreased *SPRED1* had significantly lower 2-year progression-free survival and event-free survival rates. *In vitro*, ectopic overexpression of SPRED1 leads to a decrease of extracellular signal-regulated kinase (ERK) phosphorylation, induction of apoptosis and reduction of proliferation of THP-1 cells. Our findings suggest *SPRED1* is not only a predictor of treatment response, but also an independent prognostic factor for non-APL, and targeting Ras- Mitogen-activated protein kinase (MAPK) signaling may be a promising strategy for the treatment of AML with downregulation of *SPRED1*.

## Introduction

The *SPRED1* gene is located on chromosome 15q14 and encodes SPRED1 protein, a member of the Sprouty-related protein family. The germline loss-of-function mutations of *SPRED1* result in Legius syndrome, an autosomal dominant human disorder characterized by multiple cafe'-au-lait macules, axillary freckling, learning disabilities and macrocephaly (1, 2); however, the exact mechanisms of the pathophysiology of *SPRED1* are largely unknown. A recent study demonstrates that SPRED1 interacts with neurofibromin (3), a protein encoded by *NF1*, mutation of which leads to neurofibromatosis type 1 (NF1) syndrome. It has been well-documented that NF1 is a negative regulator of Ras-MAPK signaling, which is deregulated in most cancers (4, 5). These advances suggest that SPRED1 may regulate cell proliferation, differentiation and survival by suppressing Ras-MAPK signaling (6).

Both Legius and *NF1* syndromes have various degrees of leukemia predisposition (6). A single-nucleotide polymorphism array analysis has shown that *SPRED1* deletion is frequently found in relapsed B cell acute lymphoblastic leukemia (B-ALL) (7). Moreover, a recent study suggests that SPRED1 is a tumor suppressor and is downregulated in pediatric acute myeloid leukemia (AML) (8). Nevertheless, the contribution of SPRED1 to leukemogenesis remains controversial. Several studies have reported the lack of tumorigenesis with *SPRED1* mutation in Legius syndrome (5, 9), whereas a child with neuro-cardio-facial-cutaneous (NCFC) syndrome caused by germline *SPRED1* mutation was reported to develop AML-M5 (10). We previously found the chromosomal loss of 15q involving the *SPRED1* gene in an adult AML-M5 patient using high-resolution array CGH (aCGH) (11). Amounting evidence indicates that *NF1* is a tumor suppressor gene of AML and might be a novel therapeutic target for AML (12–14). Considering the shared phenotypes of Legius and *NF1* syndromes and the physical interaction of neurofibromin with SPRED1, we hypothesize that, similar to *NF1*, the *SPRED1* gene may be downregulated in adult AML and associated with the disease progression.

In order to define the clinical features as well as the prognostic significance of *SPRED1* in leukemia, in the present study, we determined the expression of *SPRED1* in adult AML and analyzed the association of *SPRED1* expression with clinical parameters, disease status, and survival. We further investigated the proliferation and survival of AML cell lines following ectopic overexpression and silencing of SPRED1 with RNA interference.

## Materials and Methods

### Study Population

This study was approved by the Ethical Committee of the First Affiliated Hospital of China Medical University (Beijing, China). Before data collection, written informed consent was obtained from each participant. Bone marrow (BM) samples were collected from 135 patients (69 male and 66 female) with *de novo* acute leukemia (113 AMLs and 22 ALLs). Patients younger than 14 years old were excluded from this study. The median age of patients was 47 years old (range, 14–88 years). The diagnosis was made according to the French-American-British (FAB) Cooperative Group criteria (15). The BM samples were analyzed using flow immunophenotyping, conventional chromosome banding or targeted analyses (reverse transcriptase-PCR and/or FISH). Serum and BM samples of gender-balanced 37 normal donors were collected as a healthy control group (HC).

### Therapy and Follow-Up

A total of 68 AML patients, including 26 acute promyelocytic leukemia (APL) patients (M3 subtype of FAB) and 42 non-APL patients have received intensive treatment. All APL patients were included in a national 863 clinical study for APL (Clinical Trial Identifier: 2012AA02A505). The treatment for non-APL patients and the response assessment were based on Chinese guidelines described in a previous study (16). The follow-up time for the patients, calculated from the time of randomization for induction therapy, was 24 months unless death proceeded. Serum and BM samples from patients with AML at both diagnosis and complete remission (CR) were involved for analysis.

### Ficoll Gradient Separation

BM mononuclear cells (MNCs) were isolated by Ficoll-paque gradient centrifugation (TAKARA Biotechnology, Dalian, China), aliquoted into fetal calf serum (FCS) with 10% DMSO, and cryopreserved in the liquid phase of a liquid nitrogen tank.

### Preparation of RNA and Quantitative Real-Time PCR (qRT-PCR)

Liquid nitrogen-cryopreserved (liquid phase) BM mononuclear cells were rapidly thawed in 37°C water bath, washed with phosphate-buffered saline (PBS) and collected by centrifugation. Total RNA from BM MNC or cultured cells was extracted with Trizol (TAKARA Biotechnology) according to the manufacturer's instructions. Complementary DNA (cDNA) was made from 20 ng of RNA using the SuperScript III first-strand synthesis kit and oligo-dT primer (Promega) according to the manufacturer's instructions. *SPRED1* mRNA levels in BM mononuclear cells were determined using the real-time SYBRPCR amplification kit (TAKARA) with β*-ACTIN* as an internal control. Triplicate amplification reactions were done on an ABI 7500 Real-time PCR System (Applied Biosystems, Foster City, CA, USA) and the data were analyzed by the ABI 7500 system SDS software (1.4 Version). The expression value was calculated with the comparative CT method. *SPRED1* gene-specific primers: forward 5′-GATGAGCGAGGAGACGGCGAC-3′ and reverse 5′-GTCTCTGAGTCTCTCTCCACGGA-3′; β-actin: forward 5′-TGGAGATAACACTCTAAGCATAACTAAAGGT-3′ and reverse 5′-GATGTAGTTGCTTGGGACCCA-3′. All the primers were synthesized by Invitrogen. For detection of MIR126, total RNA was isolated using a mirVana miRNA isolation kit (Ambion, Austin, TX, USA). Expression levels of *MIR126* were detected using a TaqMan MicroRNA Assay (Applied Biosystems, Foster City, CA, USA) following the manufacturer's protocol.

### Immunocytochemistry

To assess the SPRED1 protein expression in leukemia blasts, immunocytochemical staining was performed by the immunoperoxidase technique using the avidin-biotin-peroxidase complex (ABC) as described (17). Cytospin smears of BM cells from AML patients with blast ratio of more than 80% and healthy controls were fixed for 3 min in formalin acetone (3%). The specimens were incubated with peroxidase blocking enzyme for 20 min, followed by incubation with a rabbit anti-human *SPRED1* antibody (Abcam, Cambridge, UK) at 4°C overnight. The next day after washing with PBS, the specimens were incubated with a biotinylated donkey anti-goat IgG secondary antibody for 30 min. After washing three times with PBS, the protein was detected using the streptavidin-peroxidase substrate. The specimens were counter-stained with hematoxylin.

### Measurement of SPRED1 Level in Serum

Serum *SPRED1* protein concentrations were determined in 26 patients with AML and 30 healthy controls using the commercial enzyme-linked immunosorbent assay (ELISA) kit (Ji Jin Chemical Technology Co., Ltd, Shanghai, China) following the manufacturer's protocol. The Optical Density (O.D.) was read at 450 nm using a microtiter plate reader. Sample concentrations were calculated by drawing standard linear regression curves.

### Fluorescence *in situ* Hybridization (FISH)

FISH analysis was performed using SPRED1 probes RP11-644D16 (15q14:36,154,798–36,372,207, SG) and RP11-477P17 (15q14:36, 375, 079–36, 541, 177, SO) (Invitrogen) on 43 cases of AMLs according the manufacturer's instructions. Two hundred interphase nuclei were screened in each case.

### Sequence Analysis of SPRED1 Mutation

The exons and the flanking areas of *SPRED1* gene were amplified by PCR using the primers as shown in STable 1. The 25 μl PCR mixture contained 50 ng of template DNA, 1X PCR buffer, 0.2 mmol/L dNTP mix, 50 ng of each primer, 2.5 mmol/L MgCl2, and 1 unit of AmpliTaq Gold (Applied Biosystems, Foster City, CA, USA). ddNTP terminator reaction was carried out with the ABI BigDye Terminator v3.1 Cycle Sequencing kit (Applied Biosystems). Sequencing products were loaded and data were collected on ABI 3130 xl genetic analyzer (Applied Biosystems). Mutations were identified via a comparison between the sample sequences and the reference sequences using Mutation Surveyor (SoftGenetics, State College, PA, USA).

### Cell Culture

Leukemia cell lines THP-1 (AML-M5), OCI-AML2 (AML-M4), OCI-AML3 (AML-M4), Kasumi-1 (AML-M2), HL-60 (AML-M2), and NB4 (AML-M3) were obtained from American Type Culture Collection (ATCC, USA) and cultured in RPMI-1640 (GIBCO, USA) supplemented with 10% FBS.

### Plasmid Constructs and Lentivirus Transduction

The Tet-pLKO-puro vector (Addgene) was used for non-silencing control or for carrying SPRED1 target shRNA. The sequence for SPRED1 shRNA were: GCAGATGACTTACAAGCAA. For SPRED1 expression, the vector was constructed by cloning human SPRED1 cDNA (NM_152594.2) into pLV-EF1a-IRES-NEO vector (Addgene) according to the standard procedure. Lentiviral packaging and transductions were carried out using standard procedure and according to manufacturer's instructions. The cells transfected with virus carrying empty vector was used as a negative control (Control group) and untreated cells were used as blank control (Blank group).

### Western Blotting

Cells were lysed on ice with RIPA buffer (Invitrogen) containing protease and phosphatase inhibitors (Sigma). Cell lysates were centrifuged at 12,000 g for 10 min at 4°C, and the supernatants were stored at −80°C. Protein quantification was performed with the BCA protein assay (Pierce). Equal amounts of protein were separated on 10% SDS polyacrylamide gel and transferred onto polyvinylidene difluoride (PVDF) membranes. Membranes were blocked with Tris-buffered saline containing 0.1% Tween 20 and 5% dried milk powder. Antibodies to SPRED1 (Abcam, Cambridge, UK), ERK, pERK (Wanleibio, Shenyang, China) and β-actin (Invitrogen) were used to detect the corresponding proteins. Signals were developed with an enhanced chemiluminescence detection system (Pierce). All western analysis were performed for three times.

### Cell Proliferation Assay

The viability of THP-1 and OCI-AML3 cells following transfection of shRNA or plasmid was measured using the Cell Counting Kit-8 (CCK-8) (Dojindo Molecular Technologies, Kumamoto, Japan). Cells transfected with shRNA or plasmid were cultured in a 96-well plate at a density of 2 × 10^4^ cells per well for 24, 48, and 72 h. At each time point, cells were incubated in 10% CCK-8 solution for an additional 2 h at 37°C. The absorbance at 450 nm (A450) was examined on a microplate reader (CANY, Shanghai, China).

### Cell Cycle and Apoptosis Assay by Flow Cytometry

THP-1 and OCI-AML3 cells following transfection of shRNA or plasmid for 48 h were harvested and re-suspended in PBS containing 2% BSA. After centrifugation at 1,000 rpm for 5 min, cells were re-suspended with 500 μl binding buffer and mixed with 5 μl Annexin V-PE. Cells were then incubated with 5 μl 7-AAD in the dark at room temperature for 5–15 min. All assays were performed in triplicate. Cell apoptosis and cell cycle distribution were assayed by flow cytometry (BD Biosciences, San Jose, CA, USA). Specific steps for flow cytometry were performed according to the manufacturer's instructions. Data were analyzed with FlowJo 10 software.

### Statistical Analysis

The relative expression (fold change) level of *SPRED1* mRNA was calculated using the 2^−ΔΔCt^ method. For comparison between two groups, we used Student's *t*-test (when the data is normally distributed) or Mann-Whitney U test (when the data is not normally distributed). Kruskal-Wallis test was used for comparison within multiple groups. For comparison within group at diagnosis vs. post-treatment, we used Paired Student's *t*-test (when the data is normally distributed) and Wilcoxon signed rank-sum test (when the data is not normally distributed). One-way ANOVA and Bonferroni multiple-comparison tests were used for comparisons in multiple groups. Correlations between *SPRED1* and *MIR126* expression were estimated with the Spearman rank-order correlation coefficient. The frequency data were compared by chi-squared test. Overall survival (OS) was calculated from diagnosis to the date of mortality. Progression-free survival (PFS) was estimated as the interval between the date of diagnosis to date of progression. Event-free survival (EFS) was measured from the date of entry into study until treatment failure, relapse from CR, or death from any cause. The Kaplan-Meier method was used to perform the survival curve and the log-rank test was used to test statistical significance. All statistical analyses were performed using SPSS. A two-tailed *p* < 0.05 was considered as statistically significant.

## Results

### Expression of SPRED1 Significantly Decreases in *de novo* AML

**Figure 1:**
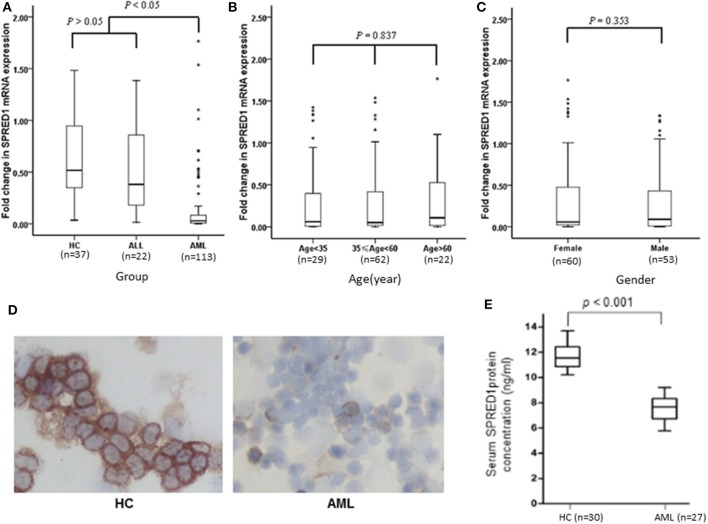
Box plots of SPRED1 mRNA expression in all subjects. SPRED1 mRNA expression **(A)** in healthy controls (HC), ALL and AML groups (Kruskal–Wallis test); **(B)** by different age groups (Kruskal–Wallis test); **(C)** between female and male (Wilcoxon tests); **(D)** representative immunocytochemical staining of SPRED1 protein in BM cells. Positive expression of SPRED1 was stained brown in BM of HC while negative expression of SPRED1 in BM of AML patient. **(E)** Serum *SPRED1* level in AML patients (7.52 ± 0.93 ng/ml) and HC (11.68 ± 0.94 ng/ml) (Wilcoxon tests).

This study included 113 AML patients, 22 ALL patients, and 37 HC persons. The characteristics of the subjects in these three groups are shown in Table 1. The mRNA expression of the *SPRED1* gene in the BM of these 172 subjects was determined by qRT-PCR. Significantly lower expression of *SPRED1* was observed in the AML group in comparison with those in the HC (*p* < 0.05) and ALL groups (*p* < 0.05). No significant difference was found between ALL and HC groups (*p* > 0.05; Figure 1A). Comparing to the HC, 99 out of 113 AMLs (87.6%) showed decreased *SPRED1* expression (defined as lower than the 25% percentile value in the HC group). We next divided the AML patients into young (age < 35), middle (35 ≤ age < 60) and old (age ≥ 60) groups and found there was no significant difference of *SPRED1* mRNA expression among these groups (*p* > 0.05; Figure 1B). In addition, there was no significant difference in *SPRED1* mRNA expression between males and females (*p* > 0.05; Figure 1C). Further analysis of SPRED1 protein of BM cells from 6 AML patients and 3 HC subjects by immunocytochemistry showed strong brown staining in the three HC subjects while four negative stainings in AML patients (Figure 1D). In addition, measurement of serum *SPRED1* level in 27 patients with AML and 30 HC using ELISA demonstrated a significantly lower *SPRED1* level in AML than HC group (*p* < 0.001; Figure 1E).

**Table 1:** Characteristics of the subjects in this study.

**Variables**	**AML group** ***n* = 113**	**ALL group** ***n* = 22**	**HC group** ***n* = 37**	***P***
**Gender**, ***n*** **(%)**				0.805
Male	53 (46.9)	12 (54.55)	18 (48.65)	
Female	60 (53.1)	10 (45.45)	19 (51.35)	
**Age (year)**	47 (34,57)	43 (27,59)	43 (31.5,52)	0.403
**Age**, ***n*** **(%)**				0.083
Age < 35	29 (25.7)	10 (47.6)	11 (30.6)	
35 ≤ Age < 59	62 (54.9)	7 (28.6)	23 (61.1)	
59 ≤ Age	22 (19.5)	5 (23.8)	3 (8.3)	
**WBC (10**^**9**^**/L)**, ***n*** **(%)**				
<10	65 (57.5)	–	–	–
≥10	48 (42.5)			
**Hb (g/L)**, ***n*** **(%)**				
≤80	61 (54.0)	–	–	–
>80	52 (46.0)			
**PLT (10**^**9**^**/L)**, ***n*** **(%) 41**				
≤50	76 (67.3)	–	–	–
>50	37 (32.7)			
**Blasts (%)**, ***n*** **(%)**				
≤72	52 (46.0)	–	–	–
>72	61 (54.0)			
**FAB subtype**, ***n*** **(%)**				
M2	31 (27.4)	–	–	–
M3 M5				
	30 (26.5) 41 (36.3)			
Other type	11 (9.7)			
**Chromosomal abnormalities**, ***n*** **(%)**				
Normal	34 (41.5)	–	–	–
t (15;17)	20 (24.4)			
Complex	8 (9.8)			
Other type	20 (24.4)			
**CR**, ***n*** **(%)**				
Yes	56 (82.4)	–	–	–
No	12 (17.6)			
**ALP**, ***n*** **(%)**				
Yes	26 (43.3)			
No	34 (56.7)			

### SPRED1 Expression Is Significantly Decreased in AML of M2 and M3 Types

**Figure 2:**
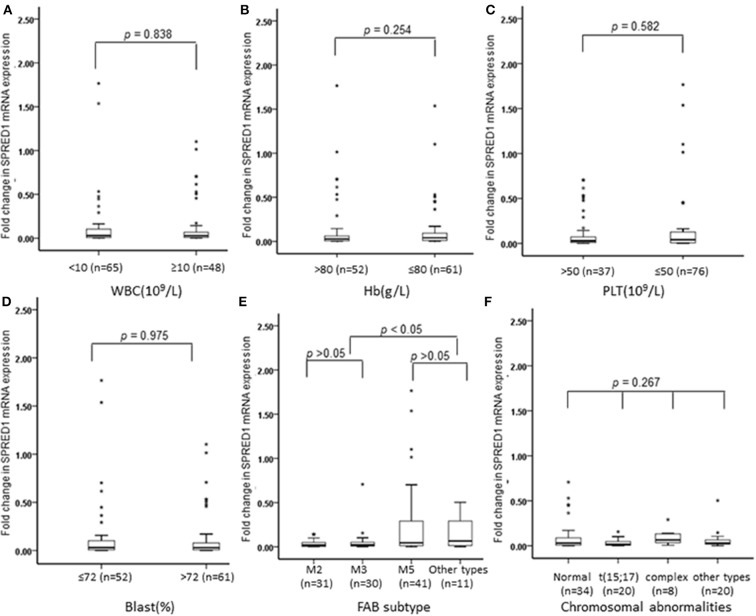
Box plots of SPRED1 mRNA expression in AML patients with different characteristics. SPRED1 mRNA expression in AML patients with different **(A)** WBC (Wilcoxon tests); **(B)** Hb (Wilcoxon tests); **(C)** PLT (Wilcoxon tests); **(D)** blast (Wilcoxon tests); **(E)** FAB subtype (Kruskal–Wallis test); **(F)** chromosomal abnormalities (Kruskal–Wallis test).

We next analyzed the correlations of *SPRED1* mRNA expression in BMs of 113 AML patients with a series of prognostic relevant clinical and laboratory parameters of AML including, white blood cell (WBC) count, hemoglobin (Hb) concentration, platelet (PLT) count and higher percent of blast cells in bone marrow (>72% comparing to the average blast ratio in bone marrow), FAB subtypes, and chromosomal abnormalities. No significant difference of *SPRED1* expressions was found between subgroups with different WBC (*p* = 0.838; Figure 2A), Hb concentration (*p* = 0.254; Figure 2B), PLT count (*p* = 0.582; Figure 2C), and the percentage of blast cells in BM (*p* = 0.975; Figure 2D). Different expression of *SPRED1* was found among the FAB subtypes of AMLs (Figure 2E). In M2, M3, and M5 subtypes with similar sample sizes, a significantly decreased expression of *SPRED1* was found in M2 and M3 compared to that of M5 and other types (*p* < 0.05). There was no significant difference of *SPRED1* expression between M2 and M3 (*p* > 0.05) and between M5 and other types (*p* > 0.05). However, no significant difference of *SPRED1* expressions was found between subgroups with different chromosomal abnormalities (*p* > 0.05; Figure 2F and STable 2).

### SPRED1 Expression Increases in AML Patients With CR After Induction Treatment

**Figure 3:**
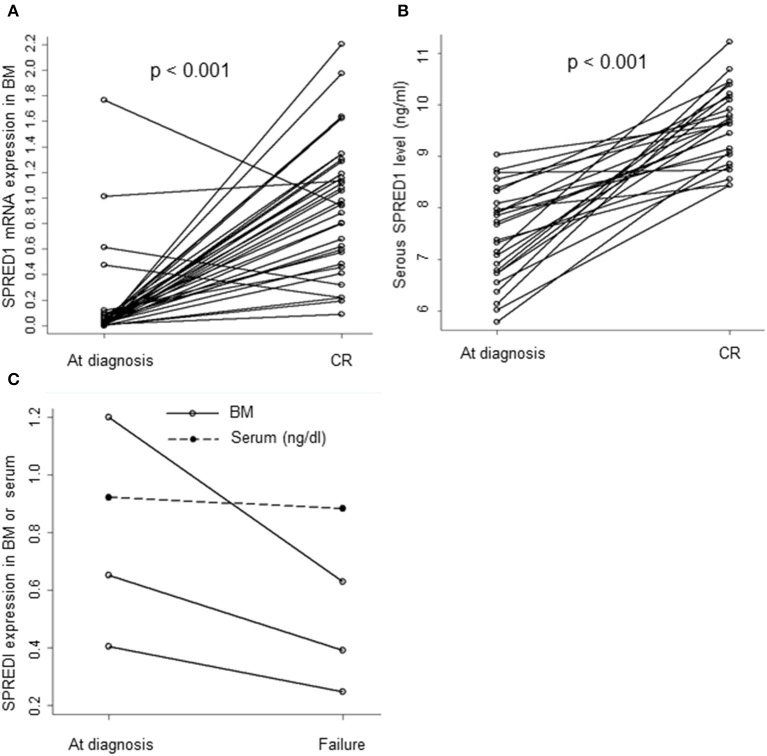
SPRED1 in AML patients at diagnosis and post-treatment. SPRED1 in AML patients with CR at **(A)** mRNA (Paired Student's *t*-test); **(B)** protein level (Paired Student's *t*-test); **(C)** SPRED1 in AML patients induction failure at diagnosis and post-treatment.

The mRNA levels in the BM samples from 37 AML patients (M0 *n* = 1, M1 *n* = 2, M2 *n* = 9, M3 *n* = 12, M5 *n* = 12, M6 *n* = 1) and serum SPRED1 protein concentrations in the serum samples from 27 AML patients were compared both at diagnosis and after induction treatment. Both the BM and the serum samples were matched. For patients with CR achievement, the median levels of *SPRED1* mRNA (0.9558 ± 0.5166, *n* = 34) and *SPRED1* protein (0.0961 ± 0.0074 ng/ml, *n* = 26) at CR were significantly higher than those at diagnosis (0.1416 ± 0.3536, 0.0745 ± 0.0091 ng/ml, all *p* < 0.001; Figures 3A,B). The majority of the patients showed increased *SPRED1* either in BM (91.9%) or in serum (96.3%) at CR. Three patients who did not achieve CR after one induction course showed significantly reduced *SPRED1* mRNA in BM (*p* = 0.044) and one of the patients had the same trend in serum (Figure 3C).

### Lower SPRED1 Expression at Diagnosis Is Associated With Worse Survival of AML Patients

**Figure 4:**
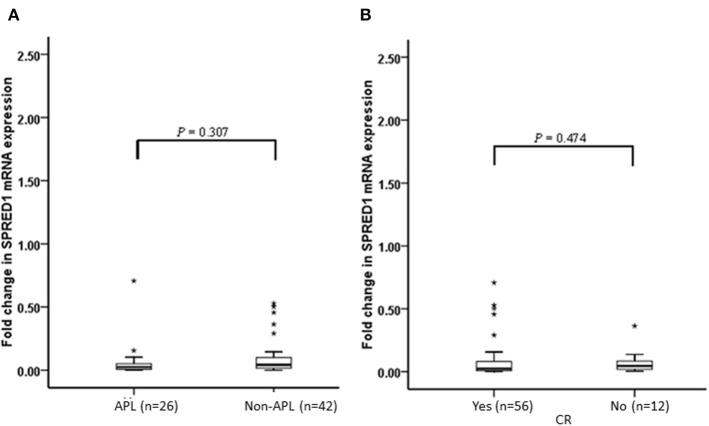
Fold change of SPRED1 mRNA expression in AML patients at follow-up. Fold change in AML patients (Wilcoxon tests); **(A)** with APL and without APL; **(B)** with and without CR (Wilcoxon tests).

**Figure 5:**
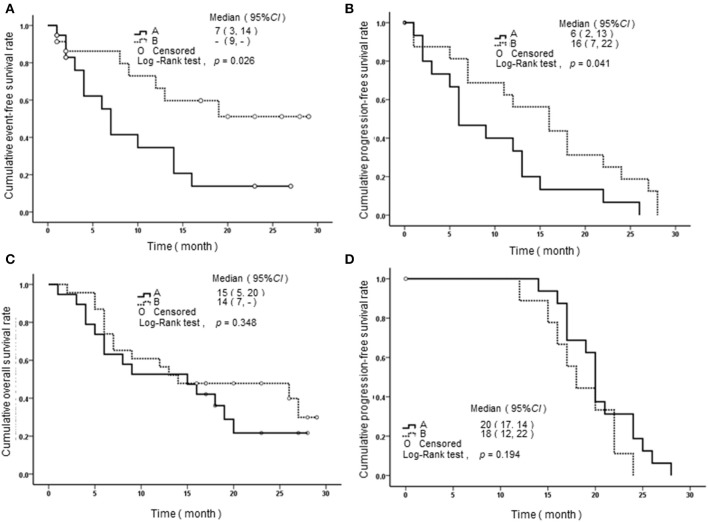
Survival curves of AML patients with different SPRED1 mRNA expression at diagnosis. **(A)** Event-free survival curves of patients with non-APL; **(B)** progression-free survival curves of patients with non-APL; **(C)** overall survival curves of patients with non-APL; **(D)** progression-Free survival curves of patients with APL. A line, fold change in SPRED1 mRNA <0.03; B line, fold change in SPRED1 mRNA ≥0.03 (log-rank test).

We performed a follow-up (median 17 months, range 2–24 months) for a total of 68 AML patients, including 26 of APL and 42 of non-APL. Fifty-six of these patients achieved CR, 25 relapsed, and 29 died. Both the non-APL patients and APL patients were further grouped based on the median of *SPRED1* expression in BM at diagnosis and rates of survival parameters were compared between the two groups. There was no significant difference in *SPRED1 mRNA* expression between APL and non-APL patients (*p* > 0.05; Figure 4A), and patients with and without CR (*p* > 0.05; Figure 4B). In non-APL, significantly lower EFS (*p* = 0.026; Figure 5A) and PFS (*p* = 0.041; Figure 5B) rate at 2 years were demonstrated in the group with lower *SPRED1* mRNA; however, there was no significant difference of OS of non-APL patients with lower or higher *SPRED1* mRNA (*p* = 0.348; Figure 5C). In APL, the group with lower *SPRED1* had comparable 2-year OS, 2-year EFS and 2-year PFS (*p* = 0.194; Figure 5D) in contrast to those of group with higher *SPRED1* expression.

### SPRED1 Gene Is Not Mutated or Deleted in *de novo* AML With Decreased SPRED1 Expression

To explore the potential mechanisms of the downregulation of *SPRED1* in AML, we performed FISH analysis of *SPRED1* in 48 AML patients, who had decreased *SPRED1* mRNA expression comparing to the healthy control. We observed that all of the cases showed normal signals in 200 interphase nuclei, suggesting no deletion of the *SPRED1* gene (SFigure 1). Furthermore, we determined the gene sequence of *SPRED1* by direct DNA sequencing in 35 of the AML patients who had a normal FISH signal. Again, we found that none of the patients carried *SPRED1* mutation.

### SPRED1 Downregulation Is Not Related to the Expression of MIR126 in AML

**Figure 6:**
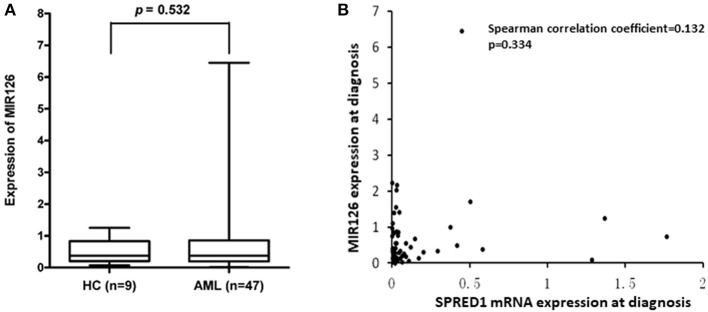
Correlation between SPRED1 and MIR126 mRNA expression level at diagnosis. **(A)** Comparison on MIR126 expression in HC and AML (Wilcoxon tests); **(B)** spearman correlation analysis between SPRED1 mRNA expression MIR126 expression.

*SPRED1* is a potential target of miR126. To test whether the reduction of SPRED1 results from the deregulation of miR126 in AML, we determined *MIR126* levels in 47 AMLs and nine controls. The *MIR126* level in AMLs (0.7321 ± 1.0318) showed a higher trend than that of the healthy controls (0.5111 ± 0.3976) with no statistical significance though (*p* = 0.532; Figure 6A). Although all except one (97.9%) of the 17 patients had decreased *SPRED1* expression, no correlation was shown between *MIR126* and *SPRED1* expression (*r* = 0.132, *p* = 0.344; Figure 6B).

### Overexpression of SPRED1 Suppresses ERK Signaling and the Proliferation of AML Cells

**Figure 7:**
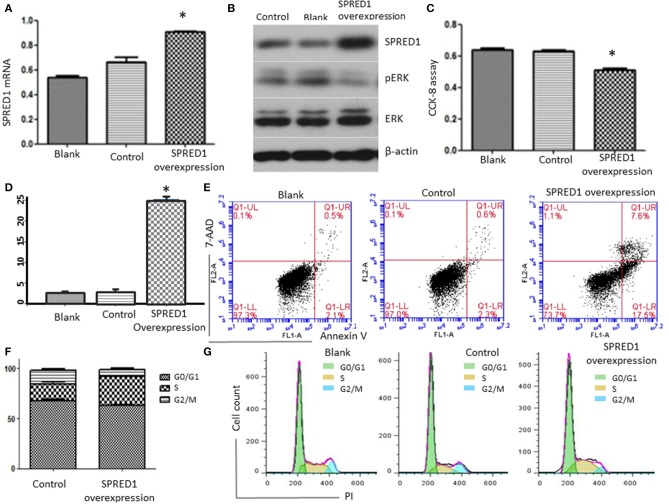
THP-1 cells were transfected with SPRED1 plasmid for 48 h. **(A)** *SPRED1* mRNA was determined by RT-PCR in the blank; **(B)** SPRED1, pERK, ERK, and β-actin) were assessed by immunoblotting; **(C)** cell proliferation was determined by CCK-8 assay; **(D,E)** cell apoptosis was assessed by using annexin V–PE/7-AAD staining and **(F,G)** cell cycle distribution was assayed by flow cytometry. *p < 0.05 vs. control (Student's *t*-test).

To assess SPRED1 in the proliferation and survival of AML cells, the mRNA expression levels of *SPRED1* in AML cell lines THP-1, OCI-AML2, OCI-AML3, Kasumi-1, HL-60, and NB4 were determined by RT-PCR. THP-1 had the lowest while OCI-AML3 had the highest *SPRED1* mRNA levels (SFigure 2A). Corresponding protein expression of SPRED1 was confirmed in cell lines THP-1, HL-60 and OCI-AML3 (SFigure 2B). In the following experiments, we used THP-1 cells to overexpress SPRED1 and OCI-AML3 cells to downregulate SPRED1. Transfection of THP-1 cells with a plasmid containing SPRED1 significantly increased the *SPRED1* mRNA level (0.91 ± 0.01) comparing to blank (0.54 ± 0.01) and control cells (0.67 ± 0.37) (*p* < 0.05; Figure 7A). Immunoblotting demonstrated that the overexpression of SPRED1 reduced the phosphorylation of ERK (Figure 7B). Compared to the empty vector, transfection SPRED1 plasmid significantly inhibited the proliferation of THP-1 cells as demonstrated by CCK-8 assay. The 450 nm optical density (OD) value in blank, control and SPRED1 overexpression cells was 0.64 ± 0.03, 0.63 ± 0.01 and 0.51 ± 0.03, respectively (*p* < 0.05; Figure 7C). The increase of apoptosis by overexpression of SPRED1 was proved by cell apoptosis assay. It showed that apoptotic cells ratio (%) in the blank, control, and SPRED1 overexpression groups was 2.73 ± 0.21, 3.00 ± 0.36, and 25.21 ± 0.59, respectively (*p* < 0.05; Figures 7D,E). Accumulation of cells at the S phase of the cell cycle in SPRED1 overexpression cells was demonstrated in Figures 7F,G. The cell ratio of G0/G1 phase, S phase and G2/M phase was 51.26 ± 5.31, 34.37 ± 4.80, and 9.76 ± 0.95% in the blank group; 67.39 ± 2.35, 16.95 ± 3.10, and 13.35 ± 2.72% in control group; 62.63 ± 2.34, 29.14 ± 1.42, and 6.07 ± 2.71% in SPRED1 overexpression group.

### Silencing of SPRED1 Activates ERK Signaling and Enhances the Proliferation of AML Cells

**Figure 8:**
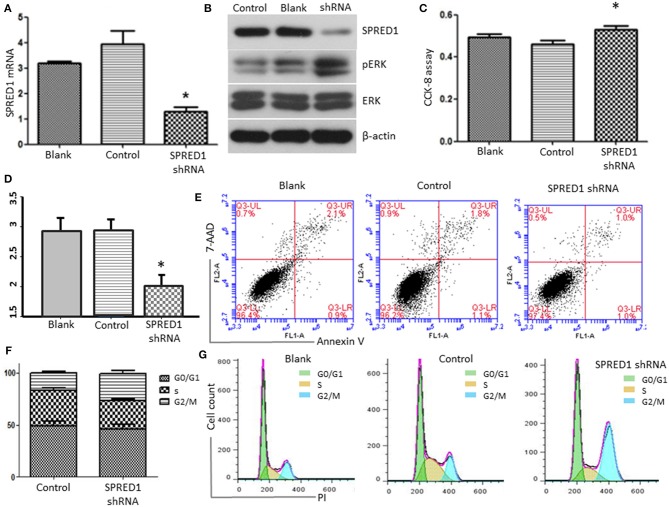
OCI-AML3 cells were transfected with SPRED1 shRNA for 48 h. **(A)** *SPRED1* mRNA was determined by RT-PCR; **(B)** SPRED1, pERK, ERK, and β-actin were assessed by immunoblotting; **(C)** cell proliferation was determined by CCK-8 assay; **(D,E)** cell apoptosis and **(F,G)** cell cycle distribution was assessed by flow cytometry. *p < 0.05 vs. control (Student's *t*-test).

Finally, we downregulated SPRED1 in OCI-AML3 cells by shRNA and assessed cell proliferation and ERK activity. Transfection of OCI-AML3 cells with a SPRED1 shRNA significantly decreased the *SPRED1* mRNA level (1.30 ± 0.17) in contrast to blank (3.19 ± 0.08) and control cells (3.95 ± 0.52) (*p* < 0.05; Figure 8A). Immunoblotting analysis demonstrated the silencing of SPRED1 by shRNA increased the phosphorylation of ERK (Figure 8B). In comparison that of scrambled control shRNA, transfection SPRED1 shRNA significantly promoted the proliferation of OCI-AML3 cells, as demonstrated by CCK-8 assay. The 450 nm optical density (OD) value in blank, control and SPRED1 shRNA cells was 0.49 ± 0.04, 0.46 ± 0.04, and 0.53 ± 0.04 (*p* < 0.05; Figure 8C). The silencing of SPRED1 by shRNA suppressed apoptosis. Apoptotic cells ratio (%) in blank, control and SPRED1 shRNA groups was 2.93 ± 0.18, 2.92 ± 0.16, and 2.01 ± 0.15 (*p* < 0.05; Figures 8D,E). The reduction of the cells at the S phase was identified by cell cycle assay. The cell ratio of G0/G1 phase, S phase and G2/M phase was 49.84 ± 6.63, 30.64 ± 8.28, and 19.74 ± 1.80% in the blank group; 49.19 ± 5.70, 33.64 ± 4.04, and 17.33 ± 1.62% in control group; 45.87 ± 6.99, 27.26 ± 2.46, and 26.25 ± 4.21% in SPRED1 shRNA group (Figures 8F,G).

## Discussion

In the present study, we demonstrated that expression of SPRED1 was significantly downregulated in AML, particularly M2 and M3 types. In a subset of non-APL patients, we observed lower SPRED1 expression at diagnosis was associated with worse PFS of AML patients. Moreover, SPRED1 expression was upregulated in AML patients with CR after induction treatment. *In vitro*, ectopic overexpression of SPRED1 suppressed ERK signaling and the proliferation of AML cells, which was accompanied the accumulation of cells at S phase and induction of cell apoptosis; in contrast, silencing of SPRED1 activated ERK signaling, enhanced the proliferation and reduced the apoptosis of AML cells.

Recent studies have suggested that *SPRED1* is attenuated, and SPRED1 is a potential tumor suppressor in pediatric ALL or AML (7, 8). However, the association of *SPRED1* expression status with the clinical features as well as the prognostic significance of *SPRED1* in acute leukemia, especially adult AML, remains to be determined. In addition, the previous study of pediatric leukemia showed that *SPRED1* expression appeared to be significantly decreased in AMLs and T-ALL, but normal in B-ALL (8). Therefore, further investigation is warranted in ALL with different immunophenotypes since most ALL cases in this study were B-ALL. Our results showed that expression of SPRED1 was significantly downregulated in adult AML, particularly M2 and M3 types. More strikingly, the decrease in SPRED1 expression was not associated with recurrent chromosomal abnormalities of AML, including *t* (8;21) and *t* (15;17), which are common in M2 and M3 (18). The cause of this contradictory result might be that the classification of chromosomal abnormalities adopted in this study was not completely consistent with the WHO classification by the limitation of the small case number of recurrent chromosomal abnormalities. Considering that *NF1* deletion frequently occurs in AML-M4eo and AML with *t* (16;16)/inv(16) (13), the association of *SPRED1* downregulation and AMLs with particular recurrent chromosomal abnormalities still needs further investigation.

The mechanism of decreased *SPRED1* expression AML is mostly unknown. Although *SPRED1* germline mutation was reported to develop AML-M5 (10), no mutation of *SPRED1* was discovered in 23 juvenile myelomonocytic leukemia (JMML) patients (19). Regarding deletion, both Olsson et al. and our group previously did detect *SPRED1* deletion with high-resolution array analysis (7, 11). In this study, however, using an equally sensitive assay of FISH analysis, we demonstrate that the deletion of *SPRED1* is a rare event in AML.

Li and his colleague proposed that *SPRED1* was the potential target of *MIR126* in AML (20). *MIR126* is entirely complementary to 3'UTR of *SPRED1* and is required for vascular integrity and angiogenesis. Recently, Lechman et al. demonstrated that *MIR126* targets the PI3K/AKT/MTOR signaling pathway, preserving leukemia stem cell quiescence and promoting chemotherapy resistance (21). Overexpression of *MIR126* can relieve the repressive influence of *SPRED1* on the signaling pathways activated by VEGF and FGF (22, 23). Prior study also reported the overexpression of *MIR126* in core-binding factor (CBF) AMLs (20). However, neither a significant elevation of *MIR126* nor its negative correlation with *SPRED1* was observed in our study. The discrepancy may come from the fact that our study focused on general AML instead of specific subgroup identified on cytogenetic abnormalities and gene mutations. As reduction of *SPRED1* occurred much more frequently than did elevation of *MIR126* in this study (87.6 vs. 36.2%) and no distinction of *SPRED1* expression was observed between CBF AML and AML with other recurrent chromosomal abnormalities, we speculate that upregulation of *MIR126* is not the major underlying mechanism of *SPRED1* reduction even if it does impact the *SPRED1* expression in certain subgroup of AML.

Currently, there is a lack of reports on the treatment response and survival duration of AML with abnormal expression of SPRED1. Our study indicates that *SPRED1* serves not only as a marker of treatment response but also as an independent prognostic factor for non-APL. We found that decreased *SPRED1* expression was significantly related to the inferior EFS and PFS of non-APL. It suggests that *SPRED1* plays a vital role in the progression of leukemia. Therefore, it is necessary to investigate the contribution of SPRED1 to leukemogenesis. SPRED1 regulates the proliferation and differentiation of hematopoietic cells, modulates angiogenesis and mediates cell metastasis (24–27). SPRED1 interacts with multiple factors such as *NF1*, c-KIT and VEGFR thereby suppressing Ras-MAPK signaling, which promotes leukemogenesis (5, 28–32). Consistent with these previous studies, we demonstrated that ectopic overexpression of SPRED1 lead to a decrease of pERK, induction of apoptosis and reduction of proliferation of THP-1 cells; in sharp contrast, silencing of SPRED1 activated ERK signaling, enhanced the proliferation and reduced the apoptosis of OCI-AML3 cells, which highly express *SPRED1*. Therefore, one of the mechanisms by which SPRED1 functions as a tumor suppressor in AML is to antagonizing Ras-MAPK signaling. However, more functional analysis in cells and animals are needed to explore the underlying mechanism of SPRED1 in AML.

## Conclusion

**Figure 9:**
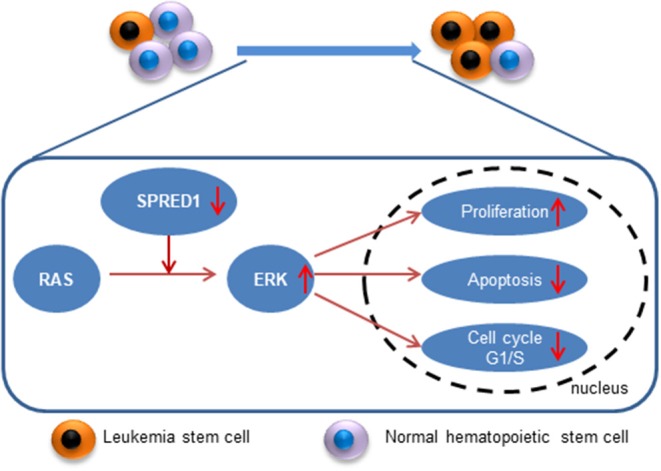
A schematic diagram of the down-regulation of SPRED1 in leukemia transformation.

*SPRED1* is downregulated in adult AML, and *SPRED1* is not only a predictor of treatment response but also an independent prognostic factor for non-APL. Mechanistically, SPRED1 may suppress AML transformation by antagonizing Ras-MAPK signaling (Figure 9). With the advance of precision medicine coupled with next-generation sequencing, targeting Ras-MAPK signaling may be a promising strategy for the treatment of AML with the downregulation of *SPRED1*.

## Data Availability Statement

The raw data supporting the conclusions of this article will be made available by the authors, without undue reservation, to any qualified researcher.

## Ethics Statement

This study was approved by Ethical Committee of the First Affiliated Hospital of China Medical University (No. AF-SOP-07-1. 0-01, date Feb 25, 2016) and conducted in accordance with the Declaration of Helsinki. The patients/participants provided their written informed consent to participate in this study.

## Author Contributions

YL designed the study. RZ and YZ prepared the manuscript. XL, WX, and HW performed the experiments. WM, HP, and RT collected the data. SL and XY made the data analysis. All the authors made the final approval.

### Conflict of Interest

The authors declare that the research was conducted in the absence of any commercial or financial relationships that could be construed as a potential conflict of interest.

## Supplementary Material

SFigure 1 FISH hybridization with home-brewed SPRED1 probes named RP11-644D16 (SG, 15q14) and RP11-477P17 (SO, 15q14). Two green signals and two orange signals were observed, suggesting no deletion of SPRED1.

SFigure 2 The expression of SPRED1 in AML cell lines. **(A)** The mRNA expression levels of SPRED1 in AML cell lines THP-1, OCI-AML2, OCI-AML3, Kasumi-1, HL-60, and NB4 were determined by RT-PCR; **(B)** the protein expression of SPRED1 in AML cell lines THP-1, OCI-AML3, and HL-60 were assayed by Western blot.

STable 1 Primers used to amplify SPRED1 exons (coding regions).

STable 2 The results of FAB subtypes, Karyotypes and FISH using SPRED1 probes for 43 *de novo* AML patients with reduced *SPRED1* expression.

## Abbreviations

SPRED1: Sprouty-Related EVH1 Domain-Containing Protein1
AML: acute myeloid leukemia
ALLs: acute lymphoblastic leukemias
non-APL: Non-acute promyelocytic leukemia
ERK: extracellular signal-regulated kinase
MAPK: Mitogen-activated protein kinase
FISH: fluorescence *in situ* hybridization
NCFC: neuro-cardio-facial-cutaneous
FAB: French-American-British
CR: complete remission
FCS: fetal calf serum
PBS: phosphate buffered saline
ELISA: enzyme-linked immunosorbent assay.
