# Supplementary figures and images for: SPRED1 Is Downregulated and a Prognostic Biomarker in Adult Acute Myeloid Leukemia
Source: Front Oncol. 2020 Feb 27;10:204. doi: 10.3389/fonc.2020.00204 (PMC7056905; doi:10.3389/fonc.2020.00204)

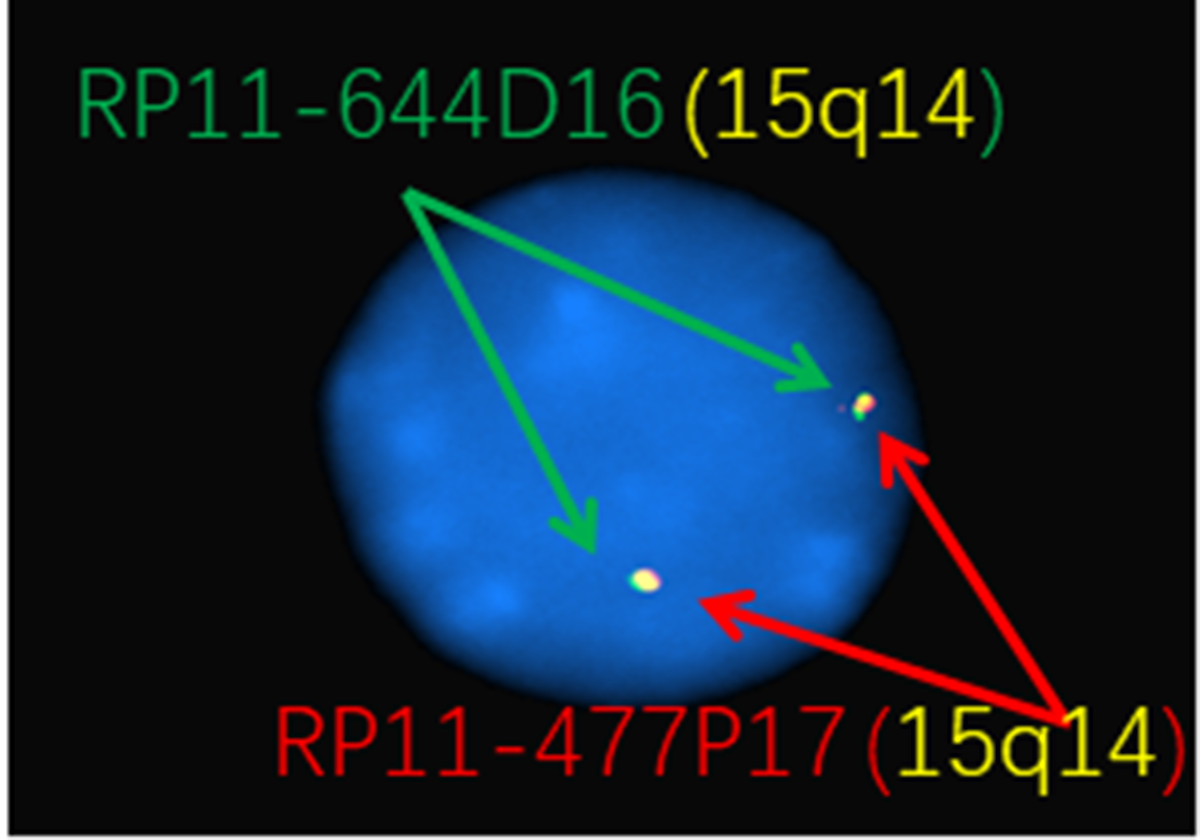

Supplement: SFigure 1 — FISH hybridization with home-brewed SPRED1 probes named RP11-644D16 (SG, 15q14) and RP11-477P17 (SO, 15q14). Two green signals and two orange signals were observed, suggesting no deletion of SPRED1. [file Image_1.TIF]

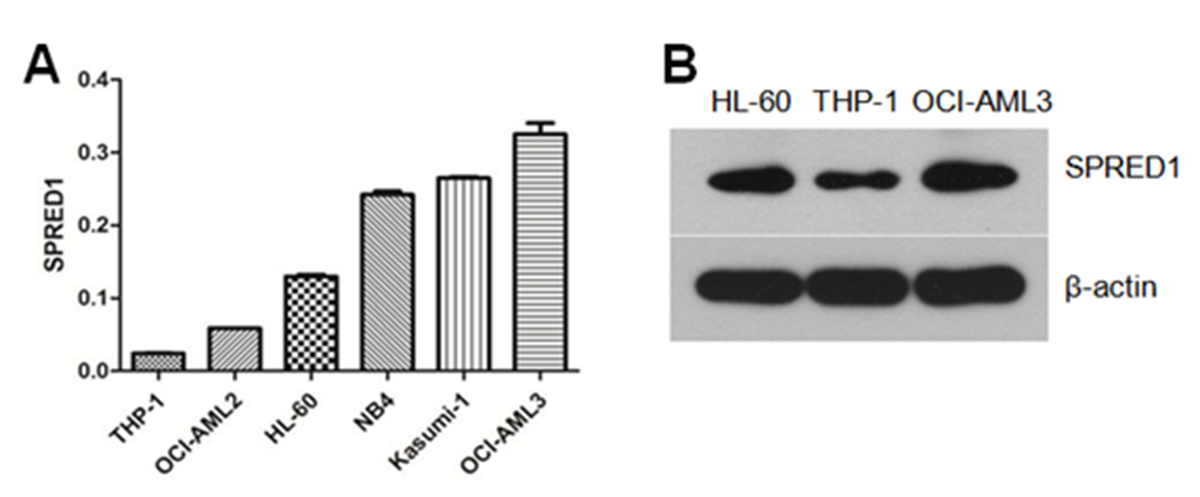

Supplement: SFigure 2 — The expression of SPRED1 in AML cell lines. (A) The mRNA expression levels of SPRED1 in AML cell lines THP-1, OCI-AML2, OCI-AML3, Kasumi-1, HL-60, and NB4 were determined by RT-PCR; (B) the protein expression of SPRED1 in AML cell lines THP-1, OCI-AML3, and HL-60 were assayed by Western blot. [file Image_2.TIF]
