# Supplementary material for: SPRED1 Is Downregulated and a Prognostic Biomarker in Adult Acute Myeloid Leukemia
Source: Front Oncol. 2020 Feb 27;10:204. doi: 10.3389/fonc.2020.00204 (PMC7056905; doi:10.3389/fonc.2020.00204)
Supplement: STable 1 — Primers used to amplify SPRED1 exons (coding regions). [file Table_1.DOCX]

STable1. Primers used to amplify SPRED1 exons (coding regions)

| Exon | Forward Primer (5'-3') | Reverse Primer (5'-3') | Size (bp) |
| --- | --- | --- | --- |
| 1 | CTGCTGTTGCTCCTCCATCT | GAAGATGCACCGAACTCTCC | 200 |
| 2 | TTTTTGGTTTTTGACCTCTTTCA | CCCCCAACAAATGTGTATCA | 549 |
| 3 | TGAGTGACTTTGTGAAGTAGACCA | TGATGTGACAAATACAAATCACG | 518 |
| 4 | TAACAGGGGCAAATGCAAGT | TTTCAGATTTTGGAACATTTCAG | 408 |
| 5 | CATTTGAGTTTTGGGAATTGCT | TTCACTCTGGATTTGGCTCA | 485 |
| 6 | TGGTTTTTGTTTGTGTTTTAGGTG | CAGCGTTTAAGCACATAGGC | 420 |
| 7-1* | GGTCCGCCTATACCACAGAA | TCACACGAACAGGGATCAGA | 693 |
| 7-2* | TTAAGACGCAGCCTTCCTCA | AACAAAACATGCCATGCACT | 601 |

Note: * Exon 7 is covered by PCR fragments yielded from 7-1 and 7-2 primer pairs.
