# Supplementary material for: SPRED1 Is Downregulated and a Prognostic Biomarker in Adult Acute Myeloid Leukemia
Source: Front Oncol. 2020 Feb 27;10:204. doi: 10.3389/fonc.2020.00204 (PMC7056905; doi:10.3389/fonc.2020.00204)
Supplement: STable 2 — The results of FAB subtypes, Karyotypes and FISH using SPRED1 probes for 43 de novo AML patients with reduced SPRED1 expression. [file Table_2.DOCX]

STable2. The results of FAB subtypes, Karyotypes and FISH using SPRED1 probes for 43 de novo AML patients with reduced *SPRED1* expression

| ID | FAB Subtype | Karyotype | FISH |
| --- | --- | --- | --- |
| 1 | M2 | 46,XX,t(7;11)(p15;p15)[16]/46,XX[4] | nuc ish(RP11-644D16 x2/RP11-477P17 x2)[200] |
| 2 | M2 | 46,XX[20] | nuc ish(RP11-644D16 x2/RP11-477P17 x2)[200] |
| 3 | M2 | 46,XY[20] | nuc ish(RP11-644D16 x2/RP11-477P17 x2)[200] |
| 4 | M2 | 45,X,-Y,t(8;21)(q22;q22)[8]/46,XY[12] | nuc ish(RP11-644D16 x2/RP11-477P17 x2)[200] |
| 5 | M2 | 46,XY[20] | nuc ish(RP11-644D16 x2/RP11-477P17 x2)[200] |
| 6 | M2 | 47,XY,-8,+?del(11),+mar[2]/46,idem,-21[4]/46,XY[14] | nuc ish(RP11-644D16 x2/RP11-477P17 x2)[200] |
| 7 | M2 | 46,XX[20] | nuc ish(RP11-644D16 x2/RP11-477P17 x2)[200] |
| 8 | M2 | 46,XX[20] | nuc ish(RP11-644D16 x2/RP11-477P17 x2)[200] |
| 9 | M2 | 46,XX,t(8;21)(q22;q22)[8]/46,XX[12] | nuc ish(RP11-644D16 x2/RP11-477P17 x2)[200] |
| 10 | M2 | 46,XX[20] | nuc ish(RP11-644D16 x2/RP11-477P17 x2)[200] |
| 11 | M2 | 45,X,-X,t(8;21)(q22;q12)[20] | nuc ish(RP11-644D16 x2/RP11-477P17 x2)[200] |
| 12 | M2 | 46,XY[20] | nuc ish(RP11-644D16 x2/RP11-477P17 x2)[200] |
| 13 | M2 | 47,XY,del(2)(p21p24),+del(2),t(8;21)(q22;q22)[20] | nuc ish(RP11-644D16 x2/RP11-477P17 x2)[200] |
| 14 | M2 | 45,X,-Y[10]/46,XY[10] | nuc ish(RP11-644D16 x2/RP11-477P17 x2)[200] |
| 15 | M2 | 46,XY[20] | nuc ish(RP11-644D16 x2/RP11-477P17 x2)[200] |
| 16 | M3 | 46,XY,t(15；17)(q22;q12)[20] | nuc ish(RP11-644D16 x2/RP11-477P17 x2)[200] |
| 17 | M3 | 46,XX,t(15;17)(q22;q12)[20] | nuc ish(RP11-644D16 x2/RP11-477P17 x2)[200] |
| 18 | M3 | 46,XY, t(15;17)(q22;q12)[20] | nuc ish(RP11-644D16 x2/RP11-477P17 x2)[200] |
| 19 | M3 | 46,XX,t(2;4)(q33;q32),t(15;17)(q22;q12)[20] | nuc ish(RP11-644D16 x2/RP11-477P17 x2)[200] |
| 20 | M3 | 46,XX, t(15;17)(q22;q12)[8]/46,XX[12] | nuc ish(RP11-644D16 x2/RP11-477P17 x2)[200] |
| 21 | M3 | 46,XX[20] | nuc ish(RP11-644D16 x2/RP11-477P17 x2)[200] |
| 22 | M3 | 46,XY, t(15;17)(q22;q12)[20] | nuc ish(RP11-644D16 x2/RP11-477P17 x2)[200] |
| 23 | M3 | 46,XY, t(15;17)(q22;q12)[20] | nuc ish(RP11-644D16 x2/RP11-477P17 x2)[200] |
| 24 | M3 | 46,XY, t(15;17)(q22;q12)[12]/46,XY[8] | nuc ish(RP11-644D16 x2/RP11-477P17 x2)[200] |
| 25 | M3 | 47,XX,+13[12]/46,XX[4] | nuc ish(RP11-644D16 x2/RP11-477P17 x2)[200] |
| 26 | M3 | 46,XY, t(15;17)(q22;q12)[8]/47,idem,+21[4]/46,XY[8] | nuc ish(RP11-644D16 x2/RP11-477P17 x2)[200] |
| 27 | M3 | 46,XY, t(15;17)(q22;q12)[4]/46,XY[16] | nuc ish(RP11-644D16 x2/RP11-477P17 x2)[200] |
| 28 | M3 | 46,XX, t(15;17)(q22;q12)[20] | nuc ish(RP11-644D16 x2/RP11-477P17 x2)[200] |
| 29 | M3 | 46,XX, t(15;17)(q22;q12)[20] | nuc ish(RP11-644D16 x2/RP11-477P17 x2)[200] |
| 30 | M3 | 46,XX[20] | nuc ish(RP11-644D16 x2/RP11-477P17 x2)[200] |
| 31 | M3 | 46,XX,?inv(9)(p11q12)[19]/46,idem,t(15;17)(q22;q12)[1] | nuc ish(RP11-644D16 x2/RP11-477P17 x2)[200] |
| 32 | M3 | 46,XY[20] | nuc ish(RP11-644D16 x2/RP11-477P17 x2)[200] |
| 33 | M3 | 46,XY t(15;17)(q22;q12)[18]/46,XY[2] | nuc ish(RP11-644D16 x2/RP11-477P17 x2)[200] |
| 34 | M5 | 46,XX,t(X;10)(p21;p12),dup(1)(q25q44)[20] | nuc ish(RP11-644D16 x2/RP11-477P17 x2)[200] |
| 35 | M5 | 46,XY[20] | nuc ish(RP11-644D16 x2/RP11-477P17 x2)[200] |
| 36 | M5 | 46,XX[20] | nuc ish(RP11-644D16 x2/RP11-477P17 x2)[200] |
| 37 | M5 | 47,XY,+?der(5)[20] | nuc ish(RP11-644D16 x2/RP11-477P17 x2)[200] |
| 38 | M5 | 47,XX,+8[16]/46,XX[4] | nuc ish(RP11-644D16 x2/RP11-477P17 x2)[200] |
| 39 | M5 | 46,XX,-22,+mar[20] | nuc ish(RP11-644D16 x2/RP11-477P17 x2)[200] |
| 40 | M5 | 46,XX,t(6;11)(q27;q23)[16]/46,XX[4] | nuc ish(RP11-644D16 x2/RP11-477P17 x2)[200] |
| 41 | M5 | 45,X,-Y[20] | nuc ish(RP11-644D16 x2/RP11-477P17 x2)[200] |
| 42 | M5 | 47,XX,+8[12]/52,idem,+10,+13,+20,+21,+mar[8] | nuc ish(RP11-644D16 x2/RP11-477P17 x2)[200] |
| 43 | M5 | 46,XX,?inv(16)(p13q22)[20] | nuc ish(RP11-644D16 x2/RP11-477P17 x2)[200] |
| 44 | M5 | 46,XY,t(9;22)(q34;q11),13p+[10]/46,idem,15q+[10] | nuc ish(RP11-644D16 x2/RP11-477P17 x2)[200] |
| 45 | M5 | 47,XY,+8[16]/47,idem,del(9)(q13q22)[4] | nuc ish(RP11-644D16 x2/RP11-477P17 x2)[200] |
| 46 | M5 | 46,XY[20] | nuc ish(RP11-644D16 x2/RP11-477P17 x2)[200] |
| 47 | M6 | 46,XX[20] | nuc ish(RP11-644D16 x2/RP11-477P17 x2)[200] |
| 48 | M6 | 44,XY,t(2;12)(p21;p13),del(5)(q13q33),del(7)(p15)[20] | nuc ish(RP11-644D16 x2/RP11-477P17 x2)[200] |
